# Facets of the Fundamental Content Dimensions: Agency with Competence and Assertiveness—Communion with Warmth and Morality

**DOI:** 10.3389/fpsyg.2016.01810

**Published:** 2016-11-22

**Authors:** Andrea E. Abele, Nicole Hauke, Kim Peters, Eva Louvet, Aleksandra Szymkow, Yanping Duan

**Affiliations:** ^1^Department of Psychology and Sport Science, University of Erlangen-NürnbergErlangen, Germany; ^2^School of Psychology, University of QueenslandBrisbane, QLD, Australia; ^3^Laboratoire de Psychologie des Cognitions, University of StrasbourgStrasbourg, France; ^4^University of Social Sciences and Humanities, Sopot CampusWarsaw, Poland; ^5^Department of Physical Education, Hong Kong Baptist UniversityHong Kong, China

**Keywords:** agency, communion, facet model, competence, assertiveness, warmth, morality, cross-cultural research

## Abstract

Agency (A) and communion (C) are fundamental content dimensions. We propose a facet-model that differentiates A into assertiveness (AA) and competence (AC) and C into warmth (CW) and morality (CM). We tested the model in a cross-cultural study by comparing data from Asia, Australia, Europe, and the USA (overall *N* = 1.808). Exploratory and confirmatory factor analyses supported our model. Both the two-factor model and the four-factor model showed good fit indices across countries. Participants answered additional measures intended to demonstrate the fruitfulness of distinguishing the facets. The findings support the model's construct validity by positioning the fundamental dimensions and their facets within a network of self-construal, values, impression management, and the Big Five personality factors: In all countries, A was related to independent self-construal and to agentic values, C was related to interdependent self-construal and to communal values. Regarding the facets, AA was always related to A values, but the association of AC with A values fell below our effect size criterion in four of the five countries. A (both AA and AC) was related to agentic impression management. However, C (both CW and CM) was neither related to communal nor to agentic impression management. Regarding the Big Five personality factors, A was related to emotional stability, to extraversion, and to conscientiousness. C was related to agreeableness and to extraversion. AA was more strongly related to emotional stability and extraversion than AC. CW was more strongly related to extraversion and agreeableness than CM. We could also show that self-esteem was more related to AA than AC; and that it was related to CM, but not to CW. Our research shows that (a) the fundamental dimensions of A and C are stable across cultures; and (b) that the here proposed distinction of facets of A and C is fruitful in analyzing self-perception. The here proposed measure, the AC-IN, may be a useful tool in this research area. Applications of the facet model in social perception research are discussed.

## Introduction

The distinction between the “fundamental dimensions” or “Big Two” of agency (A) and communion (C) is one that is pervasive in many fields of psychological theorizing and research. Our goal in the present article was to extend the A–C framework by presenting a model that differentiates these fundamental dimensions into two facets each. We also wanted to show that the resulting four facets are universal across different cultures and languages. The aim is to broaden our understanding of the A–C framework and to open new avenues of research into people's self-perceptions and their perceptions of other individuals and groups, including differences in actor-observer perceptions and stereotyping across cultures.

## The agency—communion framework

Research has consistently revealed that there are two fundamental dimensions of content—sometimes called the “Big Two”—in many fields of social cognition^1^. These are the dimensions of agency (A) and communion (C). While agentic content refers to qualities relevant for goal-attainment, such as being ambitious or capable, communal content refers to qualities relevant for the establishment and maintenance of social relationships, such as being friendly or fair. A and C capture the two recurring challenges of human life: Pursuing individual goals and belonging to social groups (Ybarra et al., 2008). Coined by Bakan (1966), those conceptual labels have provided an effective framework for the analysis of social cognition, self-perception, impression management, social perception, stereotyping, values, motives, and personality (for reviews see Paulhus and John, 1998; Judd et al., 2005; Fiske et al., 2007; Cuddy et al., 2008; Paulhus and Trapnell, 2008; Abele and Wojciszke, 2014).

The A and C conceptualization is an integrative framework for different lines of research in social, personality, motivation, and cross-cultural psychology. In the case of stereotypes (Fiske et al., 2007), the A and C framework has demonstrated, among other things, that stereotypes differ not only in valence but also in content (the stereotype content model; Fiske et al., 2007). Indeed, stereotypes may be simultaneously positive with respect to the content dimension of communion (called warmth) and negative with respect to the content dimension of agency (called competence) or vice versa. In the case of person perception (Wojciszke, 2005; Abele and Wojciszke, 2007; Abele and Bruckmüller, 2011), group perception (Leach et al., 2007; Brambilla et al., 2011, 2012), self-perception (Wojciszke et al., 2011; Gebauer et al., 2013), and actor—observer differences in impression formation (Abele and Wojciszke, 2007; Abele et al., 2014) the A and C framework has revealed that the A dimension is more important when actors perceive themselves than when they observe others. In contrast, the C dimension is more important than the A dimension when perceiving others. At the same time, the C dimension has been found to be primary, receiving higher ratings and faster recognition than A content (Ybarra et al., 2001; Abele and Bruckmüller, 2011).

The two themes of agency and communion have been shown to emerge in the autobiographical narratives of adults (Diehl et al., 2004; Uchronski, 2008) and children (Ely et al., 1998). They also emerge in research on fundamental motives and values. For instance, McAdams (1988) distinguishes between the intimacy motive (affiliation; C) and the power motive (influence, uniqueness; A); in a similar way, Hogan (1982) framed his socio-analytic theory around A and C, labeling the two primary human motives “getting ahead” (A) and “getting along” (C). Horowitz et al. (2006) even used the terms “agentic motive” (individual influence, control, or mastery) and “communal motive” (connection; participation in a larger unit with other people). When it comes to values, which can be seen as cognitive representations of basic motives or broad goals that are important to people in their lives and guide their perception, judgments, and behavior (Rokeach, 1973; Schwartz, 1992; Trapnell and Paulhus, 2012), people have been shown to differ in the degree to which they value the motives of getting ahead (A; exemplified by power, expertise, success etc.) and getting along (C; exemplified by relational obligations, “purpose” in life and sacrificing for others; Trapnell and Paulhus, 2012).

The A and C framework has been shown to map onto personality as well. In particular, the seminal work on the circumplex model (Wiggins, 1979, 1991) showed that personality can be represented with the dimensions of dominance—submissiveness (A content) and nurturance—cold-heartedness (C content). Paulhus and colleagues (Paulhus and John, 1998; Paulhus and Trapnell, 2008) extended this reasoning, using the A and C framework to distinguish between different self-presentational styles: An agentic self-presentational style tends to form a “super-hero” impression (a person presenting him/herself as being able to master every challenge), whereas a communal self-presentational style tends to form a “saint” impression (a person presenting him/herself as always acting in moral ways).

While the majority of this work has been conducted with North American and Western European samples, there is some evidence that is consistent with the possibility that the A and C dimensions are culturally universal (Abele et al., 2008c; Ybarra et al., 2008; Saucier et al., 2014).

## Facets of agency and communion

As the above analysis shows, the A and C framework has seen broad usage. Nonetheless, there are differences in the specific ways in which this framework is conceptualized in the various research traditions. These differences can be seen in the labeling of the constructs: A is also called “competence,” “masculinity,” or “instrumentality”; C is also called “warmth,” “femininity,” or “morality” (Spence et al., 1974; Fiske et al., 2002; Judd et al., 2005; Wojciszke, 2005; Abele et al., 2008c). Importantly, not all of these labels are conceptually equivalent. For instance, the specific conceptualization of “competence” (i.e., “competent,” “smart”) is somewhat different from the specific conceptualization of “masculinity” (i.e., “decisive,” “assertive”), even though both competence and masculinity form a common cluster of A. Similarly, the specific conceptualization of “warmth” (i.e., “friendly,” “empathetic”) is somewhat different from the specific conceptualization of “morality” (i.e., “fair,” “honest”), even though they form a common cluster of C (see Abele and Wojciszke, 2007, Study 1). There are also differences in the construct pairs chosen, so while Fiske et al. (2002) pair competence with warmth, Wojciszke (2005) pairs competence with morality. We suggest that the differences in the labeling, operationalization, and pairing of constructs are not spurious, but that they instead reflect the multi-faceted nature of the fundamental dimensions. The present paper addresses this multi-faceted nature of the fundamental dimensions.

We propose that A and C can be distinguished into two main facets each: In the case of A, these are assertiveness (agency assertiveness; AA) and competence (agency competence; AC); in the case of C, these are warmth (communion warmth; CW) and morality (communion morality; CM). These facets have emerged repeatedly in past work and there is preliminary evidence of their usefulness. We will now discuss the justification for these facets in more detail.

The competence-assertiveness distinction of A can be understood from the consideration that successful goal pursuit or “getting ahead” requires both ability and motivation. We suggest that competence (ability, capability) reflects the former, more ability-related component of A and assertiveness (ambition, confidence) reflects the latter, more motivation-related component of A. For example, describing a person as ambitious implies that this person is highly motivated to pursue his/her goals. Describing a person with competence-related traits, on the other hand, says less about his or her motivation. For instance, describing a person as capable implies that this person will pursue his/her goals efficiently if he/she is motivated (but does not presume that he/she is). The inspection of items used for assessing the “A” dimension also point to this distinction (for instance, “energetic,” “active,” “strong,” “ambitious,” “self-confident,” “assertive” vs. “intelligent,” “talented,” “effective,” “smart”). First empirical evidence comes from research by Carrier et al. (2014) who distinguished between assertiveness and competence and found that the ascription of status was more related to assertiveness than to competence.

We suggest that C content can be divided into content that relates more strongly to the “warmth” construct and content that relates more strongly to the “morality” construct. The establishment and maintenance of social relationships or “getting along” needs behaviors that are on one hand warm and friendly and on the other fair and reliable. While warmth pertains to being benevolent to people in ways that facilitate affectionate, cooperative relations with them, morality refers to being benevolent to people in ways that facilitate correct and principled relations with them by the adherence to ethics and important social values (Brambilla and Leach, 2014; Kervyn et al., 2015). Both warmth and morality related traits are intentional, and hence motivational, components (Fiske et al., 2002, 2007). The inspection of items used for assessing the “C” dimension point to this distinction (for instance, “fair,” “honest,” “considerate” vs. “warm,” “friendly,” “aware of feelings of others”). Empirical evidence for the distinction of C components comes from research on the evaluation of groups (see Leach et al., 2007; Brambilla et al., 2011, 2012, 2013; Brambilla and Leach, 2014; Kervyn et al., 2015). This work has shown that the morality facet of C is more important in judgments of groups than the warmth facet (Leach et al., 2007; Brambilla et al., 2011, 2012, 2013; Brambilla and Leach, 2014; Kervyn et al., 2015). Catellani and colleagues (Bertolotti et al., 2013; Catellani and Bertolotti, 2014) were the first to distinguish between four components in the judgments of politicians (i.e., competence; leadership—akin to assertiveness; warmth; and morality). They found that participants drew on all these components when forming impressions of politicians.

## Nomological network of agency and communion and their facets

A closer understanding of A and C and their facets proposed here may be achieved by integrating them into a nomological network of related constructs. We will concentrate on self-construal, values, impression management, and personality here, as researchers conceptualized those constructs either directly within the A and C framework (values: Trapnell and Paulhus, 2012; impression management: Blasberg et al., 2014) or in frameworks related to A and C (self-construal: Markus and Kitayama, 1991; personality: Wiggins, 1979, 1991; McCrae and Costa, 1996). Further, we will be concerned with self-esteem since there are somewhat inconsistent findings regarding the relation between the two fundamental dimensions and self-esteem.

### Self-construal

When defining who they are, people can use different views of the self; these different views are influenced by culture (Hofstede, 1980; Triandis, 1995). Markus and Kitayama (1991; Kitayama et al., 2015) proposed that people in western cultures hold an independent view of the self, i.e., they emphasize their separateness from others, their internal attributes, and their uniqueness. In contrast, many non-western people stress their connectedness to others, their social context, and their relationships when construing who they are, i.e., an interdependent self-concept.

### Values

Trapnell and Paulhus (2012) distinguished between agentic (A) and communal (C) values. A values refer to goals related to self-enhancement (power, influence, etc.) and economic achievement (wealth, status). C values refer to goals related to interpersonal relationships (trust, harmony, etc.).

### Impression management

Blasberg et al. (2014) defined impression management as intentional distortion of self-descriptions and differentiated between agentic and communal impression management. Agentic impression management means to present the self as a “super-hero” and communal impression management means to present the self as a “saint” (Paulhus and John, 1998; Paulhus and Trapnell, 2008).

### Personality

As has been shown in previous research, personality can also be described by two broad factors of dominance—submissiveness (related to A) and nurturance—cold-heartedness (related to C; Wiggins, 1979, 1991). However, present-day personality research is dominated by the Big Five trait conceptualization (McCrae and Costa, 1996) and we will include this conceptualization into our analyses to replicate and refine previous findings. According to the Big Five, personality is described by neuroticism or—as the positive pole—emotional stability (i.e., anxiety, angry hostility, depression, insecurity, and their opposites), extraversion (i.e., warmth, gregariousness, activity), conscientiousness (i.e., efficiency, order, dutifulness), openness to experience (i.e., fantasy, aesthetics, intellectual experiences), and agreeableness (i.e., altruism, compliance, tender-mindedness).

## Agency, communion, their facets, and self-esteem

Self-esteem, defined as the feeling of self-worth (Rosenberg, 1965), is a fundamental appraisal of the self and relates to the overall value a person places on the self. We included this construct into our research because previous findings were somewhat inconsistent regarding the impact of A and C, and because we suggest that the here proposed facets of A and C may help to understand the inconsistencies. Previous findings suggest that self-esteem is strongly related to A, and, if A is controlled for, not to C (Abele and Wojciszke, 2007; Abele et al., 2008c; Wojciszke et al., 2011). However, there are also findings showing that under specific conditions C may be relevant too (Bi et al., 2013; Gebauer et al., 2013).

## Agency and communion across cultures

Culture can be defined as the shared systems of meaning that characterize the members of particular groups and distinguish them from the members of different groups (Hofstede et al., 2010). Although researchers have described cross-national cultural differences in terms of a variety of cultural dimensions (Hofstede, 1980; Markus and Kitayama, 1991; Schwartz, 1992; Triandis, 1995; Hofstede et al., 2010; Kitayama et al., 2015), the distinction between cultures' independent vs. interdependent social orientation has received particular attention. An independent social orientation is characterized by values that focus on autonomy and individualism, on an independent self-construal, and an individual achievement motivation. An interdependent social orientation, in contrast, is characterized by values that focus on collectivism and harmony, on an interdependent self-construal, and a self-other interconnection motivation (Varnum et al., 2010; Stephens et al., 2012).

Although cultures differ in social orientation, preliminary evidence suggests that the fundamental content dimensions of A and C do emerge across cultures (Abele et al., 2008b; Ybarra et al., 2008; Bi et al., 2013; Abele, 2014; Saucier et al., 2014). To date there is, however, no evidence that the proposed distinction of AA, AC, CW, and CM can be reliably established across cultures. We expect that these facets will also emerge across cultures.

## Present research

Our first goal in the present article was to develop and test our facet-model in different cultures and languages. The resulting measure will be the *Agency-Communion-Inventory* (AC-IN) with A and C scales and with the facets of AA, AC, CW, and CM. Our second goal was to place the AC-IN into a nomological network of related constructs and to analyze the cultural stability/variability of the findings.

### Nomological network

Regarding self-construal, values, and impression management we predicted that ratings of A (C) traits should relate to an independent (interdependent) self-construal, to A (C) values, and to agentic (communal) impression management. We did not formulate specific hypotheses regarding the four facets of A and C. Instead, we tested their associations with these three measures in an exploratory fashion.

Regarding the Big Five conceptualization of personality and based on previous research (Paulhus and John, 1998; Blackburn et al., 2004; Gebauer et al., 2012) we predicted that emotional stability and conscientiousness should be related to ratings of A, whereas agreeableness should be associated with ratings of C. Extraversion comprises both A components (being energetic and forceful) and C components (being warm and friendly) and we predicted that extraversion is related to both ratings of A and C. Findings on the association of openness with A or C are currently inconclusive, and we did not formulate a hypothesis in this regard.

Regarding the proposed facets of A and C, we suggest that AA should be more strongly related to both emotional stability and extraversion than AC, whereas AC should be more strongly related to conscientiousness than AA. We derive these predictions from a comparison of the definition of the facets with the definitions of the respective Big Five factors. We further suggest that CW should be more strongly related to agreeableness than CM because being agreeable means to care about others and to be helpful. CW should also be more strongly related to extraversion than CM because the communal components of extraversion, being warm and friendly, refer to CW.

### Self-esteem

Regarding self-esteem and the A dimension, we predicted that AA should be more strongly related to self-esteem than AC. We base this prediction on the reasoning that AA is more strongly related to status than AC (Carrier et al., 2014), and that status, in turn, is positively related to self-esteem (Barkow, 1980; Gebauer et al., 2015). Concerning the C dimension, we predicted that CM should be associated with self-esteem, whereas CW should be of minor importance. We based this reasoning on sociometer theory, which considers self-esteem to be a metric of one's social acceptance (Leary, 2012), and on findings that morality is more strongly related to social acceptance than warmth (Brambilla and Leach, 2014).

### Culture

We also expected that, while there may be cross-cultural differences in the endorsement of self-construal, values, and/or A and C, the associations of A and C and their facets with the additional measures and with self-esteem should show evidence of cross-cultural consistency. Preliminary evidence for this prediction stems from research showing that A and C are similarly related to self-esteem (Bi et al., 2013) and to life satisfaction (Abele, 2014) across different cultures.

Studies 1a to 1c were conducted in Germany; Study 2 was conducted in France, Study 3 in Australia, Studies 4a and 4b in Poland, Study 5 in China, and Study 6 in the USA. According to Hofstede et al. (2010), the USA and Australia are highest on individualism (scores of 91 and 90 on a scale from 0 to 100), France (score 71), Germany (score 67), and Poland (score 60) are somewhat lower and China (score 20) is the lowest. Table 1 gives an overview of all sample characteristics with a total of *N* = 1.808 participants.

**Table 1:** **Sample characteristics**.

**Sample**	**Gender**			**Age**		**Country of residence**	**Recruitment**	**Participants**	**Reward**
	***N* (total)**	***N* (female)**	***N* (male)**	***M***	***SD***				
1a	114	93	21	21.35	5.94	Germany	Internet	University students	Course credit
1b	194	94	99	23.03	3.03	Germany	Campus	University students	Candy
1c	362	261	100	56.72	15.13	Germany	Internet/Campus/snowball sampling	University students & employees	Course credit/none
2	250	210	40	20.52	4.43	France	Internet	University students	None
3	140	103	37	22.68	4.97	Australia	Campus	University students	10 Austr. $
4a	189	149	40	26.13	6.75	Poland	Campus	University students	Course credit
4b	175	149	26	23.40	6.58	Poland	Internet	University students & employees	None
5	244	132	112	20.29	1.30	China	Campus	University students	None
6	140	86	54	25.50	9.58	USA	Snowball sampling	University students & employees	None

*Cases in which numbers do not add up are due to missing data*.

## Method

### Item selection for the AC-IN

We constructed our items for measuring agency and communion after a literature search of studies conducted in the present framework.^2^ We compiled the adjectives that were used most frequently. We chose 16 items designed for measuring A (more AA related: “ambitious,” “assertive,” “can make decisions easily,” “feel superior,” “have leadership abilities,” “independent,” “never give up easily,” “purposeful,” “self-confident,” “stand up under pressure”; more AC related: “capable,” “clever,” “competent,” “efficient,” “intelligent,” “persistent”), and 12 C items (more CW related: “affectionate,” “caring,” “empathetic,” “friendly,” “helpful,” “warm”; more CM related: “considerate,” “fair,” “honest,” “just,” “reliable,” “trustworthy”). The items were available in English and they were translated into the other languages represented here by native speakers of the respective language and retranslated by another native speaker. This translation-retranslation process was repeated until a satisfactory solution was achieved (i.e., until original and back-translated versions reached equivalent meanings; see Ægisdóttir et al., 2008). Thus, participants always rated themselves on a total of 28 agency and communion items.

### Additional measures

Participants also filled out measures of self-construal, values, impression management, and personality. The measures varied by sample. All participants, however, filled out a measure of self-esteem (Rosenberg, 1965).

### Studies 1a to 1c: Germany

#### Participants and procedures

A total of 670 individuals participated in the first set of studies (see Table 1). Participants of *Study 1a* were university students who answered an online questionnaire (consisting of A/C items, Big Five, independent/interdependent self-construal, self-esteem) and received course credit for participation. Participants of *Study 1b* were university students who answered a paper-and-pencil version of the questionnaire (consisting of A/C items, independent/interdependent self-construal, self-esteem) and received a piece of candy for participation. Participants of *Study 1c* were university students and employees (41.5% had completed secondary education and 58.5% had A-levels or a university degree). This large and heterogeneous sample was approached in several locations. 50 participants answered the questionnaire online; the others answered a paper-and-pencil version (A/C items, Big Five, self-esteem).

#### Measures

Our item pool for measuring A and C together with their two facets each comprised 28 items (see above). The items were presented in a bipolar format with a 5-point Likert-scale (e.g., very friendly—2-1-0-1-2—very unfriendly). The positive and negative traits were counterbalanced on the left or right side of the scale. Participants were asked to indicate how much the respective traits apply to them. The bipolar scales were later recoded to 1 to 5 with higher ratings representing the positive pole of the trait (i.e., “very friendly” in the above example).

Independent vs. interdependent self-construal was measured with the German version (Hannover et al., 2000) of the interdependent/independent self-construal scale (SCS; Singelis, 1994; sample items: Independent: “I'd rather say “no” directly than risk being misunderstood”; interdependent: “I have respect for the authority figures with whom I interact”). The items were answered on 7-point rating scales ranging from 1 = *strongly disagree* to 7 = *strongly agree*.

We measured the Big Five personality traits by means of a German short version of the Big-Five inventory (BFI-K; Rammstedt and John, 2005). It consists of 21 items that were answered on a 7-point scale from 1 = *does not apply to me* to 7 = *fully applies to me*. Sample items are “I am enthusiastic and can motivate others” (extraversion); “I am depressed, blue” (neuroticism); “I am generally trusting” (agreeableness).

Self-esteem was measured with a German version (von Collani and Herzberg, 2003) of the classical Rosenberg (1965) Self-Esteem Scale. It consists of 10 items (sample item “On the whole, I am satisfied with myself”). Participants answered the items on a 5-point scale from 1 = *strongly disagree* to 5 = *strongly agree* (sample 1b) or on a 7-point scale from 1 = *strongly disagree* to 7 = *strongly agree* (samples 1a and 1c). These different response formats were later transformed into a comparable one by the following formula: (original answer − 1) divided by (scale maximum − 1). This transformation was performed for all self-esteem answers in all studies described here.

### Study 2: France

Participants (see Table 1) were university students who answered the questionnaire online. They received no credit for participation. Participants completed a French version of the A/C items (same answering format as in Germany) and a Big Five personality inventory (Plaisant et al., 2010). The Big Five were measured with 8 to 10 items per scale and with a 5-point response format. Participants also answered a measure of A and C values (Trapnell and Paulhus, 2012; French translation by the present authors). The importance of 20 values^3^ was rated on a 7-point scale ranging from *not important for me* to *highly important for me*. Sample items for A values were “achievement,” “ambition,” “power,” and “influence.” Sample items for C values were “trust,” “honesty,” “harmony,” and “altruism.” Participants also completed a French version (Vallières and Vallerand, 1990) of the Rosenberg (1965) self-esteem scale (5-point response format).

### Study 3: Australia

Participants (see Table 1) were university students who came from different cultural backgrounds (*N* = 90 Australian and Western countries; *N* = 50 Asian countries). They completed an English version of the A/C items (same response format as above), the self-construal scale (SCS; Singelis, 1994; same answering-format as in Study 1), the Rosenberg (1965) self-esteem scale (5-point response-format), and the Mini-IPIP Big Five measure (Donnellan et al., 2006). The Mini-IPIP consists of 4 items per scale [examples: Extraversion “I am the life of the party”; agreeableness: “I sympathize with others' feelings”; conscientiousness: “I get chores done right away”; neuroticism: “I have frequent mood swings”; intellect/imagination (openness): “I have a vivid imagination”]. The response format was 5-point. Participants also answered the measure of A and C values (as in Study 2; Trapnell and Paulhus, 2012). The scales were part of a larger research project. Respondents answered a paper-and-pencil questionnaire and participated in exchange for AU$10. We analyzed the data from the Australian sample both as one group and separated by cultural background.

### Study 4a and 4b: Poland

Participants (see Table 1) of *Study 4a* were university students who received course credit for participation. They answered a paper-and-pencil questionnaire containing the A/C items (with the same response format as above), a Polish version of the SCS (Pilarska, 2011; same response format as above), a Polish version (Łaguna et al., 2007) of the Rosenberg (1965) self-esteem scale (4-point response-format), and a Polish translation (by the present authors) of the A and C values scale (Trapnell and Paulhus, 2012; same response format as above).

For *Study 4b*, the questionnaire was also posted on Facebook (different discussion groups) in the interest of getting a diverse sample. Study 4b, hence, comprised a mixed online sample of Polish participants who answered the A/C items (same response format) and the self-esteem scale (7-point response-format) only. They received no reward for participation.

### Study 5: China

Participants (see Table 1) were university students who answered the paper-and-pencil questionnaire in regular classes. They completed a Chinese version of the A/C items, a Chinese version of the SCS (Huang et al., 2009), a Chinese version (Leung and Wong, 2008) of the Rosenberg (1965) self-esteem scale (5-point response-format), and a Chinese translation of the A/C values questionnaire (translation by the present authors; same items as before; Trapnell and Paulhus, 2012; same response format as above).

### Study 6: USA

Participants (see Table 1) were university students and employees who answered an online questionnaire that was posted on popular internet platforms. The questionnaire contained the A/C items (same answering format as before), the measure of A and C values (same items and answering format as before; Trapnell and Paulhus, 2012), the Rosenberg (1965) self-esteem scale (5-point response-format), and the bidirectional impression management index (BIMI; Blasberg et al., 2014). The BIMI comprises two subscales designed to tap agentic and communal impression management. Participants rated 10 statements per scale on a 7-point scale ranging from 1 = *not true* to 7 = *very true*. Sample items are “My leadership of a group guarantees the group's success” (agentic impression management) and “I never swear” (communal impression management).

### Analyses

We first conducted exploratory factor analyses (EFA) using maximum likelihood analysis with the 28-item-set in order to see if the adjectives that were gathered from previous research result in factors that correspond to the fundamental dimensions and their facets. Next, we conducted multi-group confirmatory factor analyses (CFA) with the aim of deleting items with low factor loadings and selecting a 20 item set with 5 items per facet (AA, AC, CW, CM). With this resulting 20 item set, we estimated two-factorial (A and C only) and four-factorial (AA, AC, CM, and CW) models for the overall sample and also for each country separately. We also compared the fit of the two-factor models with the corresponding fit of the four-factor models.

We then tested the psychometric properties of the newly developed scales and analyzed their associations with the additional measures. As the samples of the studies that we report in this paper differed in size, we did not rely on significance testing only, but we also inspected effect sizes and shared variances. We interpreted correlations that exceeded *r* = 0.22, because variables then share at least 5% of variance. Using Cohen's (1988) guidelines, we interpreted differences that were greater than *d* = 0.30. Finally, we demonstrated the added value of the differentiation of A and C by testing the association of the facets with self-esteem by means of regression analyses.

## Results

### Exploratory factor analyses

First we conducted an EFA with all 28 A and C items with all *N* = 1.808 participants from the 6 studies reported above. Following recommendations by Costello and Osborne (2005) we used maximum likelihood method as the extraction criterion. This resulted in six factors with eigenvalues >1 (first factor: 23.3%; second factor: 11.1%; third factor: 5.0%; fourth factor: 4.6%; fifth factor: 4.0%; sixth factor: 3.9%). Altogether, the six factors explained 51.9% of the item variance.

According to the scree plot criterion (Cattell, 1966), two factors were rotated. Table 2 shows the rotated two-factor solution (Varimax rotation). It is a perfectly clear A (factor 1) vs. C (factor 2) solution without any cross-loadings >0.30. We also ran the six-factor-solution in order to see if our facets show up (Table 2). The first factor can be explained as a CW factor, the second and sixth factor can be explained as AA, the third factor as a mixture of AA and AC items, the fourth as CM, and fifth as AC.

**Table 2:** **Exploratory factor analysis for all 28 agentic and communal items (***N*** = 1.808)**.

**Extraction criterion**	**Scree plot**			**Eigenvalue** > **1**					
Factor	1	2	Factor	1	2	3	4	5	6
Explained variance	23.3%	11.1%	Explained variance	23.3%	11.1%	5.0%	4.6%	4.0%	3.9%
Self-confident	**0.69**	0.03	Warm	**0.66**	0.09	0.09	0.11	0.01	0.13
Feel superior	**0.62**	−0.07	Caring	**0.60**	0.03	0.11	0.24	0.10	−0.07
Have leadership abilities	**0.58**	0.02	Empathetic	**0.59**	0.01	0.06	0.16	0.06	0.03
Stand up under pressure	**0.58**	0.01	Friendly	**0.55**	0.09	0.10	0.19	0.04	−0.12
Capable	**0.51**	0.29	Affectionate	**0.51**	−0.09	−0.05	0.16	0.14	0.22
Efficient	**0.50**	0.28	Helpful	**0.44**	0.03	0.26	0.26	−0.02	−0.14
Clever	**0.50**	0.17	Self-confident	0.11	**0.80**	0.20	0.03	0.12	0.05
Persistent	**0.50**	0.24	Feel superior	0.02	**0.64**	0.15	−0.05	0.18	0.12
Never give up easily	**0.49**	0.22	Stand up under pressure	0.01	**0.49**	0.27	0.06	0.17	0.05
Assertive	**0.49**	0.09	Have leadership abilities	0.00	**0.42**	0.18	0.07	0.25	**0.30**
Competent	**0.47**	0.25	Persistent	0.12	0.22	**0.58**	0.15	0.08	0.05
Purposeful	**0.47**	0.24	Purposeful	0.17	0.14	**0.56**	0.05	0.16	0.13
Ambitious	**0.46**	0.21	Never give up easily	0.13	0.26	**0.55**	0.14	0.03	0.07
Can make decisions easily	**0.45**	0.01	Ambitious	0.14	0.13	**0.49**	0.04	0.24	0.11
Intelligent	**0.43**	0.16	Capable	0.10	0.18	**0.36**	0.27	**0.34**	0.17
Independent	**0.42**	0.02	Independent	−0.08	0.29	**0.33**	0.14	0.08	0.00
Caring	0.09	**0.63**	Just	0.28	0.02	0.01	**0.54**	0.16	0.08
Considerate	0.02	**0.57**	Honest	0.17	0.02	0.12	**0.52**	0.03	0.07
Empathetic	0.04	**0.57**	Fair	0.22	0.05	0.03	**0.52**	0.16	0.01
Warm	0.13	**0.56**	Reliable	0.21	0.09	0.21	**0.48**	−0.00	0.06
Trustworthy	0.18	**0.55**	Trustworthy	**0.36**	0.08	0.21	**0.41**	0.09	−0.12
Friendly	0.09	**0.53**	Considerate	**0.38**	−0.15	0.09	**0.39**	0.10	0.14
Just	0.13	**0.53**	Intelligent	0.06	0.18	0.09	0.11	**0.68**	0.04
Helpful	0.11	**0.52**	Clever	0.15	**0.31**	0.21	0.03	**0.50**	−0.04
Affectionate	0.01	**0.50**	Competent	0.10	0.18	0.21	0.22	**0.43**	0.22
Fair	0.14	**0.47**	Efficient	0.09	0.15	**0.33**	0.26	**0.38**	0.25
Reliable	0.21	**0.45**	Assertive	0.04	**0.30**	0.23	0.10	0.12	**0.46**
Honest	0.13	**0.44**	Can make decisions easily	−0.03	**0.32**	0.21	0.08	0.06	**0.34**

*Maximum likelihood analyses with Varimax rotation, loadings > |0.30| are presented in bold*.

We then conducted separate EFAs for the six countries and compared the resulting solutions. Again, the scree plot always resulted in two main factors (A and C) that explained item variances between 32.0% (Australia) and 41.4% (USA). In total, the EFAs revealed five (Poland) to eight (Australia) factors with eigenvalues >1 and the explained item variances were between 52.0% (Germany) and 64.3% (USA). We inspected these factors and found evidence for our facets of AA, AC, CW, and CM in all samples.^4^

### Item selection and model testing

#### Item selection

As we were interested in constructing scales for the four facets of A and C as explained above, we reduced the 28 item-set in such a manner that we retained those 20 items that best fit both the A and C factors and the facets of AA, AC, CW, and CM (with 5 items per facet).

We conducted a multi-group CFA using MPlus (Muthén and Muthén, 1998) in order to select the items which best fitted the four facets. The six countries (Germany, France, Australia, Poland, China, USA) served as the groups. We computed four latent variables (AA, AC, CW, and CM) initially using all 28 items. We applied a maximum likelihood estimation method. We deleted items with low primary loadings in the different group models. The solution with the best 5 items per dimension can be found in the Appendix (see Supplementary Material).

#### Model testing and comparison of the two- and four-factor model

We then computed the two-factor model of the two basic A/C dimensions as well as the four-factor model of the A/C facets. We estimated all factor loadings freely and also allowed the uniqueness of items belonging to the same subscale and having similar meaning or wording (e.g., intelligent and clever) to covary. According to our theoretical reasoning both models should exhibit good fit. We computed the models both for the overall sample and for each of the six countries separately. The fit indices for the two-factor models and for the four-factor models are shown in Table 3. The analyses revealed good model fit in all countries (cf. Fan et al., 1999; Hu and Bentler, 1999). For comparing the fit of the two-factor model with the fit of the four-factor model, AIC (Akaike Information Criterion; Akaike, 1987) and sample-size adjusted BIC (Bayesian Information Criterion; Schwarz, 1978; Tofighi and Enders, 2007; Enders and Tofighi, 2008) were used. Since lower values on these information criteria indicate better-fitting models, the model comparisons revealed that the four-factor model provided somewhat better fit for the data than the two-factor model. We conclude that both the two-factorial and the four-factorial model of A and C show cross-cultural stability.^5^

**Table 3:** **Goodness of fit indices for the two- and four-factorial A-C model**.

**Sample**	***N***	**χ^2^**	***df***	**CFI**	**TLI**	**RMSEA**	**SRMR**	**AIC**	**Sample-size adjusted BIC**
**TWO-FACTORIAL MODEL**									
Overall	1808	756.02	137	0.93	0.91	0.05	0.05	88235.55	88451.60
1 Germany	670	428.68	150	0.90	0.88	0.05	0.05	32731.10	32837.56
2 France	250	281.62	156	0.91	0.89	0.06	0.06	11636.57	11662.57
3 Australia	140	267.08	163	0.85	0.83	0.07	0.08	6671.54	6656.65
4 Poland	364	346.29	144	0.91	0.88	0.06	0.07	18353.89	18416.20
5 China	244	276.47	159	0.92	0.90	0.06	0.06	10802.78	10826.02
6 USA	140	316.81	163	0.84	0.81	0.08	0.09	6246.91	6232.02
**FOUR-FACTORIAL MODEL**									
Overall	1808	606.94	132	0.95	0.93	0.05	0.04	**88096.48**	**88324.14**
1 Germany	670	384.16	145	0.92	0.89	0.05	0.05	**32696.58**	**32809.69**
2 France	250	269.69	151	0.91	0.89	0.06	0.06	**11634.64**	**11662.39**
3 Australia	140	230.44	158	0.90	0.88	0.06	0.07	**6644.90**	**6628.90**
4 Poland	364	313.10	139	0.92	0.90	0.06	0.06	**18334.01**	**18396.63**
5 China	244	262.00	154	0.93	0.91	0.05	0.06	**10798.32**	**10823.19**
6 USA	140	250.43	158	0.90	0.88	0.07	0.08	**6190.53**	**6174.53**

*CFI = Comparative Fit Index; TLI = Tucker-Lewis Index; RMSEA = Root Mean Square Error of Approximation; SRMR = Standardized Root Mean Square Residual; AIC = Akaike Information Criterion; BIC = Bayesian Information Criterion. Modell comparison: Smaller respective AIC and BIC values presented in bold*.

### Psychometric properties

Overall and country-level means, standard deviations, reliabilities (Cronbach's alpha), and inter-correlations of the A and C scales as well as the AA, AC, CW, and CM subscales are reported in Table 4.

**Table 4:** **Characteristics of the AC-IN scales**.

**ALL PARTICIPANTS (***N*** = 1.808)**					
**Scale**	***M* (*SD)***	**α**	***r* with AC**	***r* with CW**	***r* with CM**
Agency scale (A)	3.63 (0.61)	0.82	*r* A–C = 0.33^***^		
Communion scale (C)	4.71 (0.53)	0.81			
Agency/assertiveness (AA)	3.45 (0.77)	0.74	0.55^***^	0.17^***^	0.19^***^
Agency/competence (AC)	3.81 (0.61)	0.74		0.29^***^	0.40^***^
Communion/warmth (CW)	4.13 (0.63)	0.75			0.56^***^
Communion/morality (CM)	4.21 (0.57)	0.70			
**STUDIES 1A TO 1C: GERMANY (***N*** = 670)**					
Agency scale (A)	3.69 (0.54)	0.77	*r* A–C = 0.22^***^		
Communion scale (C)	4.22 (0.63)	0.80			
Agency/assertiveness (AA)	3.61 (0.79)	0.68	0.50^***^	0.13^**^	0.10^**^
Agency/competence (AC)	3.81 (0.56)	0.67		0.22^***^	0.25^***^
Communion/warmth (CW)	4.17 (0.62)	0.76			0.54^***^
Communion/morality (CM)	4.26 (0.57)	0.66			
**STUDY 2: FRANCE (***N*** = 250)**					
Agency scale (A)	3.24 (0.57)	0.80	*r* A–C = 0.27^***^		
Communion scale (C)	4.14 (0.49)	0.79			
Agency/assertiveness (AA)	2.98 (0.84)	0.75	0.45^***^	0.14^*^	0.16^*^
Agency/competence (AC)	3.58 (0.54)	0.75		0.26^***^	0.34^***^
Communion/warmth (CW)	4.04 (0.62)	0.71			0.57^***^
Communion/morality (CM)	4.24 (0.49)	0.66			
**STUDY 3: AUSTRALIA (***N*** = 140)**					
Agency scale (A)	3.74 (0.58)	0.81	*r* A–C = 0.26^**^		
Communion scale (C)	4.16 (0.45)	0.74			
Agency/assertiveness (AA)	3.73 (0.71)	0.68	0.56^***^	0.10	0.22^*^
Agency/competence (AC)	3.85 (0.67)	0.80		0.13	0.44^***^
Communion/warmth (CW)	4.09 (0.57)	0.65			0.44^***^
Communion/morality (CM)	4.23 (0.49)	0.65			
**STUDIES 4A AND B: POLAND (***N*** = 364)**					
Agency scale (A)	3.61 (0.68)	0.84	*r* A–C = 0.35^***^		
Communion scale (C)	4.15 (0.58)	0.83			
Agency/assertiveness (AA)	3.34 (0.95)	0.79	0.56^***^	0.17^**^	0.17^**^
Agency/competence (AC)	3.85 (0.64)	0.73		0.40^***^	0.45^***^
Communion/warmth (CW)	4.14 (0.69)	0.77			0.63^***^
Communion/morality (CM)	4.17 (0.59)	0.71			
**STUDY 5: CHINA (***N*** = 244)**					
Agency scale (A)	3.59 (0.55)	0.83	*r* A–C = 0.54^***^		
Communion scale (C)	4.08 (0.49)	0.80			
Agency/assertiveness (AA)	3.48 (0.70)	0.67	0.55^***^	0.36^***^	0.41^***^
Agency/competence (AC)	3.68 (0.60)	0.82		0.43^***^	0.52^***^
Communion/warmth (CW)	4.11 (0.52)	0.71			0.60^***^
Communion/morality (CM)	4.05 (0.56)	0.68			
**STUDY 6: USA (***N*** = 140)**					
Agency scale (A)	4.05 (0.50)	0.80	*r* A–C = 0.41^***^		
Communion scale (C)	4.28 (0.58)	0.86			
Agency/assertiveness (AA)	4.03 (0.60)	0.71	0.48^***^	0.15	0.36^***^
Agency/competence (AC)	4.29 (0.58)	0.77		0.33^***^	0.54^***^
Communion/warmth (CW)	4.17 (0.70)	0.80			0.59^***^
Communion/morality (CM)	4.39 (0.59)	0.79			

* *p < 0.05*.

** *p < 0.01*.

*** *p < 0.001*.

#### Reliabilities and correlations of the AC-IN scales

In the overall sample, the A and C scales showed good reliability and had a weak positive correlation. The reliabilities for the subscales were satisfactory. The within dimension correlations (AA & AC, CW & CM) were significant and of medium effect size, *r*s > 0.54. The cross-dimension correlations (AA & CW, AA & CM, AC & CW, AC & CM) were significantly weaker, all *z*s > 6.08, all *p*s < 0.001.

The country level findings were by and large similar. The reliabilities of the A and C scales were good to satisfactory, and the reliabilities of the subscales were satisfactory. The correlations of A with C were in many cases lower than the correlations within the dimensions. Cross-dimension correlations between facets of A and C were highest for AC with CM and lowest for AA with CW. The scale intercorrelations were generally highest in China.^6^

#### Differences between the AC-IN scale means

Since repeated-measurement ANOVAs showed that there were differences regarding the level of A and C in the overall sample, *F*_(1, 1807)_ = 1208.37, *p* < 0.001, η^2^ = 0.40, as well as in the different countries, all *F*s > 21.40, *p*s < 0.001, η^2^s > 0.13, we calculated repeated-measurement *t*-tests. Regarding the overall sample, the mean of the C scale was higher than the mean of the A scale, *t*_(1807)_ = 34.76, *p* < 0.001, *d* = 1.31. The country level comparisons also showed higher endorsement of C than of A, *t*s > 4.63, *p*s < 0.001, *d*s > 0.40. These results support previous findings of a higher endorsement of C than A (Ybarra et al., 2008; Abele and Bruckmüller, 2011; Abele and Wojciszke, 2014) across cultures.

Also concerning the facets, repeated-measurement ANOVAs showed that there were differences regarding the level of AA, AC, CW, and CM in the overall sample, *F*_(3, 5421)_ = 784.63, *p* < 0.001, η^2^ = 0.30, as well as in the different countries, all *F*s > 37.81, *p*s < 0.001, η^2^s > 0.21. Thus, we calculated repeated-measurement *t*-tests to compare the means of AA and AC and of CM and CW, respectively. Regarding the overall sample, AC was higher than AA, *t*_(1807)_ = 22.15, *p* < 0.001, *d* = 0.56, but CW and CM did not differ according to our effect size criterion, *t*_(1.807)_ = 6.04, *p* < 0.001, *d* = 0.14. In all countries, AC was higher than AA, *t*s > 4.10, *p*s < 0.001, *d*s > 0.36, except for China (same direction, but lower than our effect size criterion), *t*_(243)_ = 4.51, *p* < 0.001, *d* = 0.29. CM was higher than CW in the US and in France, *t*s > 4.36, *p*s < 0.001, *d*s > 0.37. CM was also higher than CW in Germany, China, and Australia, *t*s > 2.97, *p*s < 0.01, but the effect sizes were below our criterion, *d*s < 0.26. There were no differences between CW and CM in Poland, *t*_(363)_ = 1.10, *ns*.^7^, ^8^

### Associations with related constructs

Country-level means, standard deviations, reliabilities for the additional measures, and their bivariate correlations with the AC-IN scales are shown in Table 5.

**Table 5:** **Correlations of the AC-IN scales with self-construal, values, impression management, big five personality traits, and self-esteem**.

**Measure**	***M (SD)***	**α**	**A**	**C**	**AA**	**AC**	**CM**	**CW**
**STUDIES 1A TO 1C: GERMANY (***N*** = 670, BIG FIVE: ***N*** = 476; SELF CONSTRUAL ***N*** = 308)**								
Independent self^a^	4.85 (0.68)	0.61	0.46^***^	0.18^***^	0.45^***^	0.33^***^	0.19^**^	0.12^*^
Interdependent self^a^	4.30 (0.69)	0.61	−0.17^**^	0.34^***^	−0.17^**^	−0.13	0.26^***^	0.33^***^
Neuroticism^a^	3.81 (1.29)	0.75	−0.40^***^	−0.06	−0.47^***^	−0.19^***^	−0.10^**^	0.00
Extraversion^a^	4.82 (1.24)	0.78	0.35^***^	0.23^***^	0.37^***^	0.22^***^	0.08	0.31^***^
Agreeableness^a^	4.51 (1.22)	0.67	−0.03	0.31^***^	−0.02	−0.03	0.24^***^	0.31^***^
Conscientiousness^a^	5.28 (0.97)	0.61	0.39^***^	0.40^***^	0.34^***^	0.33^***^	0.33^***^	0.37^***^
Openness^a^	5.26 (1.04)	0.69	0.20^***^	0.19^***^	0.13^**^	0.24^***^	0.14^**^	0.20^***^
Self-esteem^c^	0.76 (0.17)	0.87	0.56^***^	0.32^***^	0.55^***^	0.41^***^	0.30^***^	0.27^***^
**STUDY 2: FRANCE** ***(N*** = **250)**								
Agentic values^a^	4.29 (0.81)	0.75	0.36^***^	0.03	0.31^***^	0.29^***^	0.03	0.02
Communal values^a^	5.84 (0.65)	0.76	0.05	0.47^***^	−0.02	0.10	0.47^***^	0.39^***^
Neuroticism^b^	3.24 (0.79)	0.83	−0.55^***^	−0.07	−0.56^***^	−0.30^***^	−0.05	−0.07
Extraversion^b^	3.05 (0.81)	0.87	0.48^***^	0.30^***^	0.57^***^	0.26^***^	0.15^*^	0.36^***^
Agreeableness^b^	3.53 (0.48)	0.66	0.05	0.46^***^	−0.02	0.11	0.38^***^	0.43^***^
Conscientiousness^b^	3.41 (0.53)	0.67	0.38^***^	0.16^*^	0.26^***^	0.38^***^	0.26^***^	0.05
Openness^b^	3.44 (0.66)	0.80	0.34^***^	0.17^**^	0.27^***^	0.36^***^	0.17^**^	0.14^*^
Self-esteem^c^	0.55 (0.20)	0.88	0.67^***^	0.09	0.68^***^	0.45^***^	0.11	0.05
**STUDY 3: AUSTRALIA** ***(N*** = **140)**								
Independent self^a^	4.78 (0.73)	0.67	0.41^***^	0.25^**^	0.36^***^	0.34^***^	0.25^**^	0.17^*^
Interdependent self^a^	4.87 (0.78)	0.73	−0.14	0.25^**^	−0.10	−0.10	0.13	0.28^***^
Agentic values^a^	4.55 (0.97)	0.83	0.32^***^	−0.07	0.33^**^	0.21^*^	0.02	−0.13
Communal values^a^	5.97 (0.69)	0.81	0.01	0.42^***^	−0.05	0.10	0.35^***^	0.35^***^
Neuroticism^b^	2.82 (0.76)	0.53	−0.30^***^	−0.02	−0.28^**^	−0.17^*^	−0.10	0.06
Extraversion^b^	3.05 (0.71)	0.68	0.29^***^	0.27^**^	0.37^***^	0.12	0.16	0.29^***^
Agreeableness^b^	3.95 (0.65)	0.71	0.13	0.47^***^	0.16	0.11	0.28^**^	0.50^***^
Conscientiousness^b^	3.47 (0.70)	0.64	0.17^*^	0.15	0.19^*^	0.09	0.12	0.14
Openness^b^	3.70 (0.72)	0.70	0.25^**^	0.19^*^	0.21^*^	0.19^*^	0.15	0.17^*^
Self-esteem^c^	0.70 (0.17)	0.85	0.67^***^	0.11	0.67^***^	0.49^***^	0.24^**^	−0.03
**STUDIES 4A AND B: POLAND** ***(N*** = **364; SELF CONSTRUAL and VALUES:** ***N*** = **189)**								
Independent self^a^	5.05 (0.75)	0.73	0.52^***^	0.21^**^	0.48^***^	0.38^***^	0.15^*^	0.23^**^
Interdependent self^a^	4.21 (0.85)	0.77	−0.11	0.30^***^	−0.17^*^	0.01	0.27^***^	0.28^**^
Agentic values^a^	4.76 (1.05)	0.87	0.29^***^	−0.06	0.28^***^	0.20^**^	−0.04	−0.08
Communal values^a^	5.60 (0.85)	0.84	0.18^*^	0.56^***^	0.06	0.25^***^	0.50^***^	0.53^***^
Self-esteem^c^	0.66 (0.20)	0.90	0.70^***^	0.35^***^	0.64^***^	0.61^***^	0.33^***^	0.31^***^
**STUDY 5: CHINA** ***(N*** = **244)**								
Independent self^a^	4.87 (0.67)	0.64	0.41^***^	0.29^***^	0.34^***^	0.35^***^	0.28^***^	0.24^**^
Interdependent self^a^	5.31 (0.67)	0.64	0.16^*^	0.39^***^	0.12	0.21^**^	0.36^***^	0.33^***^
Agentic values^a^	4.65 (1.07)	0.85	0.26^***^	0.25^***^	0.24^**^	0.22^**^	0.23^***^	0.23^**^
Communal values^a^	5.50 (0.92)	0.88	0.25^***^	0.50^***^	0.22^***^	0.25^***^	0.47^***^	0.42^***^
Self-esteem^c^	0.63 (0.12)	0.76	0.62^***^	0.33^***^	0.59^***^	0.52^***^	0.36^***^	0.23^***^
**STUDY 6: USA** ***(N*** = **140; IMPRESSION MANAGEMENT:** ***N*** = **124; SELF-ESTEEM:** ***N*** = **135)**								
Agentic values^a^	4.54 (1.08)	0.87	0.21^*^	0.05	0.15	0.19^*^	−0.01	0.09
Communal values^a^	5.86 (0.82)	0.84	0.09	0.58^***^	0.04	0.13	0.51^***^	0.53^***^
Agentic IM^a^	3.42 (0.74)	0.63	0.41^***^	−0.07	0.39^***^	0.24^**^	0.03	−0.15
Communal IM^a^	3.23 (0.92)	0.69	0.01	0.06	0.08	−0.11	0.08	0.03
Self-esteem^c^	0.70 (0.19)	0.87	0.57^***^	0.22^*^	0.58^***^	0.38^***^	0.34^***^	0.07
**OVERALL FINDINGS FOR SELF-ESTEEM** ***(N*** = **1.803)**								
Self-esteem^c^	0.68 (0.19)	0.87	0.64^***^	0.28^***^	0.63^***^	0.48^***^	0.28^***^	0.21^***^

a *Scale from 1 to 7*.

b *scale from 1 to 5*.

c since original response formats differed (4, 5, or 7-point), we calculated proportional scale values (from 0 to 1) as described in the method section;

* *p < 0.05*.

** *p < 0.01*.

*** *p < 0.001; A = Agency; AA = Agency Assertiveness; AC = Agency Competence; C = Communion; CM = Communion Morality; CW = Communion Warmth; IM = impression management*.

#### Self-construal

The hypotheses regarding self-construal were supported. In all countries, A was related to independent self-construal, and C was related to interdependent self-construal. These findings are stable across cultures and the patterns were similar for the facets of A (AA and AC) and C (CW and CM; exception: In Australia, CM did not correlate with interdependent self-construal, *r* = 0.13, *ns*).

#### Values

Supporting our hypotheses, A was related to A values and C was related to C values. This association was quite stable across countries. The pattern was the same for both facets of C, but there were slight differences between the facets of A. AA was always related to A values, but the association of AC with A values fell below our effect size criterion in four of the five countries (Australia, Poland, China, USA). It seems that the association between AA and A values is somewhat higher than the association of AC with A values. The difference is, however, not significant, all *z*s < 1.57, all *p*s > 0.11.

#### Impression management

Supporting our hypothesis, A (both AA and AC) was related to agentic impression management. Unexpectedly, C (both CW and CM) was neither related to communal nor to agentic impression management.

#### Personality

The hypotheses about the association of A and C with personality were also supported. A was always related to emotional stability (negative correlation with neuroticism), to extraversion, and to conscientiousness. C was always related to agreeableness and to extraversion. Further, A was not related to agreeableness and it marginally related to openness. C was neither related to neuroticism nor to openness. Only in Germany was C related to conscientiousness. The noteworthiness of these cross-cultural findings is increased by the fact that we used different measures of personality across countries.

The predictions on the facets of A and C were also mainly supported. AA was more strongly related to emotional stability (Germany: *z* = 6.45, *p* < 0.001; France: *z* = 4.49, *p* < 0.001; Australia: *z* = 1.42, *p* = 0.08) and extraversion (Germany: *z* = 3.39, *p* < 0.001; France: *z* = 5.31, *p* < 0.001; Australia: *z* = 3.24, *p* < 0.01) than AC. CW was more strongly related to extraversion (Germany: *z* = 5.34, *p* < 0.001; France: *z* = 3.71, *p* < 0.001; Australia: *z* = 1.49, *p* = 0.07) than CM; in Germany (*z* = 1.67, *p* < 0.05) and Australia (*z* = 2.72, *p* < 0.01), it was also more strongly related to agreeableness than CM (France: *z* < 1). Only in France (*z* = 1.93, *p* < 0.05), AC was more strongly related to conscientiousness than AA (Germany: *z* < 1; Australia: *z* = −1.26, *ns*).

### Association of the facets of A and C with self-esteem

Overall and country-level means, standard deviations, and reliabilities for self-esteem, and its bivariate correlations with the AC-IN scales are shown in Table 5. As self-esteem served as a criterion measure here, we also ran stepwise linear regression analyses with a significance level of *p* < 0.05 as inclusion criterion. We chose the stepwise procedure, since we had clear predictions on the impact of the two factors (A more than C) and the four facets (AA, AC, CM, CW). The first set of regressions was computed for A and C, the second for the facets. The upper part of Table 6 displays the results of these stepwise linear regression analyses with A and C as predictors both in the overall sample and in the individual countries. Supporting previous findings (Abele et al., 2008a; Wojciszke et al., 2011; Bi et al., 2013; Gebauer et al., 2013, 2015), A always predicted self-esteem, interestingly, C also predicted self-esteem, both overall and in Germany and Poland (see also Bi et al., 2013; Gebauer et al., 2013). The negative association of C with self-esteem in France can be interpreted as a suppressor effect.

**Table 6:** **The basic dimensions and the four facets of A and C regressed upon self-esteem (stepwise linear regressions with a significance level of ***p*** < 0.05 as inclusion criterion)**.

	**Overall**	**Germany**	**France**	**Australia**	**Poland**	**China**	**USA**
**BASIC DIMENSIONS**							
A	0.61^***^	0.52^***^	0.70^***^	0.67^***^	0.66^***^	0.62^***^	0.57^***^
C	0.08^***^	0.20^***^	(−0.10^*^)	–	0.12^**^	–	–
*R*^2^ korr.	0.41^***^	0.35^***^	0.46^***^	0.44^***^	0.50^***^	0.38^***^	0.32^***^
**FACETS**							
AA	0.53^***^	0.46^***^	0.59^***^	0.57^***^	0.44^***^	0.43^***^	0.52^***^
AC	0.14^***^	0.12^**^	0.17^**^	0.19^*^	0.30^***^	0.25^***^	–
CM	0.13^***^	0.17^***^	–	–	0.11^**^	–	0.17^*^
CW	–	0.09^*^	–	–	–	–	–
*R*^2^ korr.	0.43^***^	0.37^***^	0.47^***^	0.47^***^	0.50^***^	0.38^***^	0.35^***^

* *p < 0.05*.

** *p < 0.01*.

*** *p < 0.001; significant negative β-values in brackets are suppression effects (no significant corresponding correlations) and not to be interpreted content-related; A = Agency; AA = Agency Assertiveness; AC = Agency Competence; C = Communion; CM = Communion Morality; CW = Communion Warmth*.

The bottom part of Table 6 displays the results of the stepwise linear regression analyses with the four facets of A and C as predictors. The overall findings support the prediction that AA is the best predictor for self-esteem and more strongly related to self-esteem than AC (Barkow, 1980; Carrier et al., 2014; Gebauer et al., 2015). But as predicted, CM is also related to self-esteem. At the level of the individual countries, AA is always a better predictor of self-esteem than AC even though AC has predictive value for self-esteem in all countries except for the USA. CM is a significant predictor for self-esteem in Germany, Poland, and the US, as well as in the Asian subsample from Australia (β = 0.30, *p* < 0.01; no differences regarding the other three facets). CW is a significant predictor only in Germany. The differentiation of the facets, hence, shows that AA is more strongly related to self-esteem than AC and that CM (except in France, China, and the Non-Asian Australian subsample) is also related to self-esteem but CW is not.^9^

## Discussion

### The facet model

Our goal in the present article was to present a facet model of the fundamental dimensions. This model presents AA and AC as important facets of A and CW and CM as important facets of C. The model and the resulting AC-IN will allow researchers to develop a more precise understanding of the relative importance of the different A and C components in social cognition; it will also allow a clearer understanding of differences and similarities in the importance of these components across cultures. Most importantly, it should open new avenues for research into self-perception, impression formation, actor—observer differences, group perception, and stereotyping.

EFAs of the initial 28-item set clearly revealed the fundamental content dimensions in all countries studied; and they also showed that A and C can be distinguished into the facets postulated here. We then applied CFAs to select the best-performing 20 items and to test our model. The two-factor model provided a satisfactory fit for the data, suggesting that the fundamental dimensions of A and C are stable across the cultures tested here. The analysis, therefore, provides further evidence that A and C are fundamental and universal dimensions of social judgment across languages and cultures (see Abele et al., 2008b; Ybarra et al., 2008). The four-factor model provided a better fit for the data than the two-factor model.

The scale characteristics are good to satisfactory. A and C were moderately positively correlated. This finding may be interpreted such that principally orthogonal dimensions (like A and C) correlate somewhat positively if both dimensions are rated with respect to the own person (and not with respect to the abstract construct; for a more thorough discussion of this issue see Abele and Wojciszke, 2014). The correlations between their respective facets (AA & AC; CW & CM) were generally higher than the correlations across the A and C facets (AA & CW; AC & CW; AA & CM; AC & CM). The strongest cross-dimensional correlation was consistently observed between AC and CM. This may, on the surface, appear curious given that competence and morality are sometimes treated as orthogonal dimensions (Skowronski and Carlston, 1987). We interpret this finding as reflecting the fact that, at least in self-perception, AA and CW represent the most distinct aspects of “getting ahead” vs. “getting along” (Hogan, 1982). AC and CM seem to be constructs that bridge the extremes of “getting ahead” and “getting along.”

### Network of related constructs

A showed the highest and most consistent correlations with emotional stability, extraversion, independent self-construal, agentic values, and agentic impression management. C showed the highest and most consistent correlations with agreeableness, extraversion, interdependent self-construal, and communal values. The differences between A and C are particularly evident for emotional stability, agreeableness, interdependent self-construal, agentic and communal values, and agentic impression management.

AA appears to be the core component of agency—at least when it comes to self-perception. Assertiveness captures the motivational aspect of goal-pursuit as well as a person's tendency to be self-confident and to have leadership skills. In line with these ideas, AA was most highly related to independent self-construal and to emotional stability. AA was also most strongly related to agentic impression management. Given the evidence for the importance of AA in judgments of agency, it is notable that existing theorizing and research in the agency—communion framework has paid little attention to this facet (Carrier et al., 2014; Catellani and Bertolotti, 2014).

AC was most strongly related to independent self-construal and conscientiousness. In comparison with AA, AC had weaker correlations with emotional stability and extraversion. But in contrast to AA, AC showed significant correlations with communal values in some of the samples. Consequently, while AC clearly belongs to the A domain, it also shows some connection to the C domain.

CW was most strongly related to interdependent self-construal, to communal values, to agreeableness, and also to extraversion. CM showed high associations with communal values. It differed from CW in being less related to extraversion and agreeableness. Both constructs, CM and CW, are highly salient in present research on, for instance, group perception (Brambilla et al., 2013) and stereotypes (Fiske et al., 2002, 2007). Whereas, they are clearly distinct constructs in these last-mentioned research areas, they are more strongly related in self-perception.

While the A facets were strongly related to A impression management, as expected, there was no correlation between the C facets and C impression management. A possible interpretation for this pattern of findings is that A means—among other things—striving for one's goals, and goal striving may involve impression management, like, for instance, presenting the self as the best-suited candidate for a certain job. C, in contrast, means—among others—friendliness, empathy, and fairness. Being friendly, empathic, and fair, however, implies presenting an authentic version of the self, rather than intentionally distorting this impression.

### Association with self-esteem

Whereas the above findings provide the first evidence of how the facets of A and C differ, the data on self-esteem clearly demonstrate the fruitfulness of the differentiation by showing that the distinction between facets of A and C allows a better understanding of the association between the fundamental dimensions and self-esteem. Whereas previous research has suggested that self-esteem is primarily associated with A and not with C (Abele et al., 2008a; Wojciszke et al., 2011; see also Bi et al., 2013; Gebauer et al., 2013), we could elaborate on these findings. Regarding A, it is especially the AA facet that relates to self-esteem. This finding is in line with a dominance perspective on self-esteem advanced by Barkow (1980) and supported by Gebauer et al. (2015). Most important, whereas previous research suggested that C barely relates to self-esteem, we could show that it is CW, specifically, that does not relate to self-esteem. CM, in contrast, does show associations with self-esteem. We interpret this last-mentioned finding referring to sociometer theory, which considers self-esteem to be a metric of one's social acceptance (Leary, 2012), and to findings that morality is more strongly related to social acceptance than warmth (Brambilla and Leach, 2014).

### Cross-cultural comparison

The countries analyzed here differed in their degree of independence/interdependence as indicated by Hofstede et al. (2010). Our findings on self-construal were in accord with the classification of Hofstede and colleagues.^10^ We nevertheless found more similarities than differences across the different countries and cultures. The most important similarity pertains to the fact that we could establish the same measure of our four facets of A and C in all countries analyzed here. There are also more similarities than differences in the associations of the AC-IN with the further measures taken. We conclude that the A-C framework shows stable cross-cultural associations with social orientation and personality, and that the similarities across cultures outweigh their differences.

The findings pertaining to self-esteem were also similar between countries for three of the four facets. Cultural differences were evident in the CM facet, which was a significant predictor in Germany, Poland, the USA, and in the Asian subsample from Australia, but not in France, China, and the non-Asian Australian sample.

### Implications for future research

As an integrated and cross-culturally validated framework, the AC-IN will inform a better understanding of the role of the facets of agency and communion in self-perception than the one that is afforded by a consideration of the broad dimensions of A and C only. The present research already demonstrated the fruitfulness of the approach for the analysis of self-esteem. Future research should explore under which conditions self-esteem is not only associated with AA, but also with CM, and how the differences between cultures with respect to the association between self-esteem and CM can be explained. The present research also adds to the literature on self-presentation styles, for instance, the “super-hero” vs. “saint” distinction advanced by Paulhus and John (1998). We suggest that the association of the A vs. C facets with these self-presentation styles should receive further attention. The present data suggest that the “super-hero” (i.e., a person high in A) does use agentic impression management tools, but the “saint” (i.e., a person high in C) neither uses agentic nor communal impression management tools.

It is also important to move beyond a selective consideration of the facets as this may lead to theoretically misspecified tests. For instance, it is possible that findings that have been attributed to AC or CW may in fact be driven by (unmeasured) AA or CM. Similarly, the use of an overarching framework in different areas of social judgment should help to elucidate similarities and differences in the role of the facets across these areas—such as differences in self-perception and the perception of others (“actor— observer differences”).

While we have argued for the generality of the facet approach for social judgment research, it is important to recognize that the data that we present here relates only to judgments of the self. There are, however, good reasons for expecting that our proposed model will apply to judgments of other individuals and groups. In particular, previous research has shown that the proposed facets of C, and those of A, can be empirically distinguished in research on group judgments and judgments of others as well (see Brambilla et al., 2011, 2012; Carrier et al., 2014; Kervyn et al., 2015). However, to date, this work has not examined the A and C facets together, which means that further research is needed to test the facet-model for judgments of others.

The potential utility of the facet-model can be seen in the domains of stereotypes, actor-observer effects, gossip, and reputational concern. In the case of stereotypes, warmth and competence have attracted most of the research attention; this is particularly evident from the stereotype content model (Fiske et al., 2002). It is indeed possible that warmth and competence (in the present model CW and AC) are the most important perceptual dimensions in stereotyping and that morality and assertiveness (CM and AA) have little importance^11^. However, this remains to be seen. It is also possible that different facets (e.g., AA) assume importance in some contexts (e.g., intergroup conflict) but not in others, and that they play different roles in the assessment of ingroups and outgroups. There is also evidence that there are differences in the construal of behaviors of actors (agents) and observers (recipients). According to the Dual Perspective Model (Abele and Wojciszke, 2007, 2014), actors/agents tend to construe their behavior more in terms of agency (how they achieve their goals or master a situation), whereas observers and recipients of an act construe the behavior more in terms of communion (why the actor behaved like that; what his/her intentions were). Focusing on the A and C facets may yield further insights into the nature of these differences of perspective. In a similar way, research into gossip (Peters and Kashima, 2014) and reputational challenge (Ybarra et al., 2012) have focused on morality and competence; it would be worth attending to warmth and assertiveness here, too.

## Concluding remarks

In this article, we have proposed that the fundamental dimensions of agency and communion can be distinguished into two facets each: Assertiveness and competence in the case of agency, and warmth and morality in the case of communion. We constructed a suitable measure for the self-assessment of these constructs and validated it in six different cultures. Our aim throughout this paper was to further develop the fruitful and integrative framework of agency-communion in the fields of personality and social psychology in a cross-cultural perspective. Many research questions can be analyzed with the fundamental dimensions' distinction of A and C. Some research questions may, however, be better analyzed if the facets proposed here are also applied.

## Ethics statement

All studies reported in this paper have been performed according to APA ethical standards for the treatment of human subjects. Since data collection was anonymously and involved no identifying information, in Germany, France, Poland, China, and the USA no informed consent was needed. In Australia, ethical clearance for the study was provided by the Behavioral and Social Sciences Ethical Review Committee (BSSERC) of the University of Queensland and all participants received a written participant information sheet and provided written informed consent before taking part in the study.

## Author contributions

AA and NH were responsible overall and share first authorship for this article. All authors were responsible for data collection. AA, NH, EL, and KP made substantial contributions to the conception of the work, revised it critically, and finally approved the version to be published. All authors agreed to be accountable for all aspects of the work.

### Conflict of interest statement

The authors declare that the research was conducted in the absence of any commercial or financial relationships that could be construed as a potential conflict of interest.

## Footnotes

^1^The term “content” refers to the fact that these dimensions are built with respect to semantic meaning, e.g., classes of content that have similar meaning in language.

^2^These articles were: (Wojciszke, 1994; Wojciszke et al., 1998, 2009; Fiske et al., 2002, 2007; Walker and Henning, 2004; Judd et al., 2005; Leach et al., 2007; Abele et al., 2008b; Uchronski, 2008; Wojciszke and Abele, 2008; Kervyn et al., 2009, 2015; Ames et al., 2010; Bergsieker et al., 2010, 2012; Brambilla et al., 2011, 2012; Cislak, 2013; Durante et al., 2013; Carrier et al., 2014).

^3^We used an abbreviated version with 10 A values and 10 C values. We did not include “excitement,” “pleasure” (A values), “humility” and “tradition” (C values). Trapnell and Paulhus (2012) also suggested that the scale may be shortened by omitting these items.

^4^The complete data for the six EFAs is available on request from the corresponding author.

^5^Since the model shows acceptable fit indices in all samples, the findings can be interpreted as achieving configural measurement invariance. We also tested for metric and for scalar invariance. Partial metric invariance could be shown, but scalar invariance could not be shown. However, in practical research, full measurement invariance is barely achieved and is also considered as being scientifically unrealistic (Horn et al., 1983; Horn, 1991; Steenkamp and Baumgartner, 1998). The test of measurement invariance is available on request from the corresponding author.

^6^As the Australian sample also comprised Asian participants, we tested if the scale-intercorrelations differed between the sub-samples of Non-Asian vs. Asian participants. This was not the case, all *z*s < 1.50, all *p*s > 0.13.

^7^Concerning the differences between the Australian subsamples, the Asian subsample had lower A scores (*M* = 3.54, *SD* = 0.62) than the Non-Asian one (*M* = 3.85, *SD* = 0.53), *t*_(138)_ = 3.14, *p* < 0.01, *d* = 0.55, which was due to AC (Asian participants, *M* = 3.56, *SD* = 0.74; non-Asian participants, *M* = 4.01, *SD* = 0.57), *t*_(138)_ = 4.05, *p* < 0.001, *d* = 0.71; but not to AA, *t*_(138)_ = 1.49, *ns*. The Asian subsample also had lower C scores (*M* = 4.05, *SD* = 0.48) than the Non-Asian one (*M* = 4.21, *SD* = 0.42), *t*_(138)_ = 2.03, *p* < 0.05, *d* = 0.36, which was due to CM (Asian participants: *M* = 4.05, *SD* = 0.50; Non-Asian participants: *M* = 4.32, *SD* = 0.46), *t*_(138)_ = 3.23, *p* < 0.01, *d* = 0.57; but not to CW, *t* < 1. We also compared the two Australian subsamples separately. The Asian sample had higher means in C than in A, *t*_(49)_ = 5.22, *p* < 0.001, *d* = 0.92, but the facets did not differ, *t*s < 1. The non-Asian Australian sample showed the same pattern as the complete Australian sample with the difference of CM vs. CW achieving an effect size above our criterion (*d* > 0.30), *t*_(89)_ = 3.68, *p* < 0.001, *d* = 0.43.

^8^We also tested for associations with age and gender. The responses were not correlated with age, all *r*s < 0.22 (below our effect size criterion). The correlations with gender were also low. We found gender differences only for C and only in Germany (women had higher means in C, *r* = 0.26, *p* < 0.001, especially CW, *r* = 0.28, *p* < 0.001, than men) and in the US (women had higher means in C, *r* = 0.24, *p* < 0.001).

^9^It may be argued that AA is most strongly related to self-esteem because the scale contains the item “self-confident.” Even if the Rosenberg scale (1965) measures self-esteem in a different manner, we nevertheless tested this reasoning by omitting “self-confident” from the AA scale. The findings revealed that the association of AA with self-esteem became lower, but it remained the strongest association and the pattern of findings with the other three facets remained equivalent.

^10^Participants from Poland, *t*_(188)_ = 10.70, *p* < 0.001, *d* = 1.05, and Germany, *t*_(307)_ = 10.18, *p* < 0.001, *d* = 0.80, revealed more independent than interdependent self-construal, non-Asian participants from Australia did not differ (independent self-construal *M* = 4.79, *SD* = 0.70; interdependent self-construal *M* = 4.74, *SD* = 0.80), *t* < 1, but participants with Asian background (independent self-construal *M* = 4.76, *SD* = 0.78; interdependent self-construal *M* = 5.10, *SD* = 0.71), *t*_(49)_ = *2.55, p* = 0.01, *d* = 0.36, and participants from China, *t*_(243)_ = 9.24, *p* < 0.001, *d* = 0.66, showed more interdependent than independent self-construal.

^11^It should also be noted that the selection of traits used in the stereotype content model is a broad one and resembles our A and C factors. Nevertheless, the distinction of the here proposed facets might give further insight into the content of stereotypes toward different groups.
