# Supplementary material for: Facets of the Fundamental Content Dimensions: Agency with Competence and Assertiveness—Communion with Warmth and Morality
Source: Front Psychol. 2016 Nov 22;7:1810. doi: 10.3389/fpsyg.2016.01810 (PMC5118442; doi:10.3389/fpsyg.2016.01810)
Supplement: Supplementary file 1 [file DataSheet1.docx]

*Appendix: AC-IN Items in the Different Languages*

|  | **English** | **German** | **French** | **Chinese** | **Polish** |
| --- | --- | --- | --- | --- | --- |
| **CW** | little caring – very caring | wenig fürsorglich – sehr fürsorglich | peu prévenant - très prévenant | 完全不关心 – 非常关心 | mało troskliwy/a – bardzo troskliwy/a |
|  | very cold in relations with others – very warm in relations with others | sehr kühl in Beziehungen zu anderen – sehr herzlich in Beziehungen zu anderen | très froid dans les relations aux autres - très chaleureux dans les relations aux autres | 人际关系冷漠 – 人际关系良好 | bardzo chłodny/a w relacjach z innymi – bardzo serdeczny/a w relacjach z innymi |
|  | little empathetic – very empathetic | wenig einfühlsam – sehr einfühlsam | peu empathique - très empathique | 完全没有同情心 – 非常有同情心 | mało empatyczny/a – bardzo empatyczny/a |
|  | little affectionate - very affectionate | wenig liebevoll - sehr liebevoll | peu affectueux - très affectueux | 没有感情的 - 非常有感情的 | bardzo nieczuły/a - bardzo czuły/a |
|  | not at all friendly – very friendly | sehr unfreundlich – sehr freundlich | très antipathique - très sympathique | 一点也不友善 –非常友善 | bardzo niemiły/a – bardzo miły/a |
| **CM** | unjust - just | ungerecht - gerecht | injuste - juste | 不正直 - 正直 | niesprawiedliwy/a - sprawiedliwy/a |
|  | little fair - very fair | wenig fair - sehr fair | peu équitable - très équitable | 不公正 - 非常公正 | nigdy nie jestem fair - zawsze jestem fair |
|  | very inconsiderate - very considerate | sehr rücksichtslos - sehr rücksichtsvoll | pas du tout attentionné très attentionné | 一点都不善解人意 - 非常善解人意 | zupełnie niezważający/a na innych - bardzo zważający/a na innych |
|  | not trustworthy – very trustworthy | nicht vertrauenswürdig – sehr vertrauenswürdig | pas du tout digne de confiance - tout à fait digne de confiance | 不值得信赖 – 非常值得信赖 | niegodny/a zaufania – godny/a zaufania |
|  | not very reliable - very reliable | wenig zuverlässig - sehr zuverlässig | peu fiable - très fiable | 非常不可靠 - 非常可靠 | nigdy nie można na mnie polegać - zawsze można na mnie polegać |
| **AA** | not at all self-confident – very self-confident | nicht selbstsicher – sehr selbstsicher | pas du tout sûr de soi - très sûr de soi | 完全不自信 – 非常自信 | zupełnie niepewny/a siebie – bardzo pewny/a siebie |
|  | go to pieces under pressure – stand up well under pressure | kann Druck nicht standhalten – kann Druck gut standhalten | incapable de résister à la pression - résiste bien à la pression | 抗压力弱 – 抗压力强 | słabo radzę sobie będąc pod presją – dobrze radzę sobie będąc pod presją |
|  | give up very easily – never give up easily | gebe leicht auf – gebe nie leicht auf | abandonne facilement - n‘abandonne pas facilement | 很容易放弃 – 从不轻易放弃 | poddaję się łatwo – nie poddaję się łatwo |
|  | have no leadership abilities at all - have leadership qualities | bin kein Führungstyp - habe Führungseigenschaften | leader – pas leader | 没有领导能力 - 有领导能力 | W ogóle nie jestem typem przywódcy - Jestem typem przywódcy |
|  | feel very inferior – feel very superior | fühle mich unterlegen – fühle mich überlegen | me sens inférieur - me sens supérieur | 感觉低人一等 – 有优越感 | bardzo uległy/a – bardzo dominujący/a |
| **AC** | little efficient - very efficient | wenig effizient - sehr effizient | peu efficace - très efficace | 没有效率 - 非常有效率 | mało skuteczny/a - bardzo skuteczny/a |
|  | little capable - very capable | wenig leistungsfähig - sehr leistungsfähig | peu capable - très capable | 非常机智 - 非常不机智 | bardzo niesprawny/a - bardzo sprawny/a |
|  | little competent - very competent | wenig kompetent - sehr kompetent | peu compétent - très compétent | 考虑不周全 - 考虑非常周全 | bardzo niekompetentny/a - bardzo kompetentny/a |
|  | little intelligent - very intelligent | wenig intelligent - sehr intelligent | peu intelligent - très intelligent | 没有智慧 - 非常有智慧 | mało inteligentny/a - bardzo inteligentny/a |
|  | little clever – very clever | wenig clever – sehr clever | peu ingénieux - très ingénieux | 很不聪明 – 非常聪明 | mało sprytny/a – bardzo sprytny/a |
